# Supplementary material for: Synthesis and Biological Evaluation of Novel Ramalin Derivatives as Multi-Target Agents for Alzheimer’s Disease
Source: Molecules. 2025 May 2;30(9):2030. doi: 10.3390/molecules30092030 (PMC12073177; doi:10.3390/molecules30092030)
Supplement: Supplementary file 1 [file molecules-30-02030-s001.zip › molecules-3561251-supplementary.pdf]

molecules

MDPI

Supporting information

Synthesis, Biological Evaluation of Novel Ramalin Derivatives as Multi-Target Agents for Alzheimer's Disease

Tai Kyoung Kim [1,†], Ju-Mi Hong [2,†], Yongeun Cho [3], Yeji Jeon [3], Heewon Cho [3], Jeongmi Lee [3], Jaewon Kim [2,4], Kyung Hee Kim [2,5], Il-Chan Kim [2], Se Jong Han [2], Hyuncheol Oh [6], Dong-Gyu Jo [3,6,7,8,*] and Joung Han Yim [1,2,*]

1 CRYOTECH Inc., 2F-211-3, 71 Mieumsandan 5-ro 41beon-gil, Gangseo-gu, Busan 46744, Republic of Korea; tkkim@cryotech.co.kr (T.K.K.); jhyim@kopri.re.kr (J.H.Y)
2 Division of Polar Life Sciences, Korea Polar Research Institute, Incheon 21990, Republic of Korea; wnal5555@kopri.re.kr (J.-M.H.); ashcercle@kopri.re.kr (J.K.); kh313@kopri.re.kr (K.H.K.); ickim@kopri.re.kr (I.-C.K.); hansj@kopri.re.kr (S.J.H.); jhyim@kopri.re.kr (J.H.Y.)
3 School of Pharmacy, Sungkyunkwan University, Suwon 16419, Republic of Korea; okcho9307@naver.com (Y.C.); jeonyeji183@gmail.com (Y.J.); hwcho1012@gmail.com (H.C.); jungmileedy@naver.com (J.L.); jodg@skku.edu (D.-G.J.)
4 Department of Plant Biotechnology, Korea University, Seoul 02841, Republic of Korea
5 Department of Chemistry, Hanseo University, Seosan 31962, Republic of Korea
6 College of Pharmacy, Wonkwang University, Iksan 54538, Republic of Korea; hoh@wcu.ac.kr (H.O.)
7 Samsung Advanced Institute for Health Science and Technology, Sungkyunkwan Univiesity, Seoul 06351, Republic of Korea
8 Biomedical Institute for Convergence, Sungkyunkwan University, Suwon 16419, Republic of Korea
9 Institute of Quantum Biophysics, Sungkyunkwan University, Suwon 16419, Republic of Korea
† These authors contributed equally to this work.
* Correspondence: jodg@skku.edu (D.-G.J.); Tel.: +82-31-290-7776; jhyim@kopri.re.kr (J.H.Y); Tel.: +82-32-760-5540; Fax: +82-32-760-5509

Context ....page

N[5]*-((2-methoxyphenyl)amino)-L-glutamine* (**RA-2OMe**)

Figure S1. ^{1}H NMR spectrum of **RA-2OMe** 4

Figure S2. ^{13}C NMR spectrum of **RA-2OMe**.. 4

Figure S3. HRESIMS spectrum of **RA-2OMe**.. 5

N[5]*-((4-methoxyphenyl)amino)-L-glutamine* (**RA-4OMe**)

Figure S4. ^{1}H NMR spectrum of **RA-4OMe** 5

Figure S5. ^{13}C NMR spectrum of **RA-4OMe**.. 6

Figure S6. HRESIMS spectrum of **RA-4OMe**.. 6

N[5]*-((2-(trifluoromethyl)phenyl)amino)-L-glutamine* (**RA-2CF3**)

Figure S7. ^{1}H NMR spectrum of **RA-2CF3** 7

Figure S8. ^{13}C NMR spectrum of **RA-2CF3**.. 7

Figure S9. HRESIMS spectrum of **RA-2CF3** 8

N[5]*-((4-(trifluoromethyl)phenyl)amino)-L-glutamine* (**RA-3CF3**)

Figure S10. ^{1}H NMR spectrum of **RA-3CF3** 8

Figure S11. ^{13}C NMR spectrum of **RA-3CF3**.. 9

Figure S12. HRESIMS spectrum of **RA-3CF3**.. 9

N[5]*-((4(trifluoromethoxyl)phenyl)amino)-L-glutamine* (**RA-4OCF3**)

Figure S13. ^{1}H NMR spectrum of **RA-4OCF3** 10

Figure S14. ^{13}C NMR spectrum of **RA-4OCF3** 10

Figure S15. HRESIMS spectrum of **RA-4OCF3**.. 11

N[5]*-((naphtahlen-1-ylamino)-L-glutamine* (**RA-NAP**)

Figure S16. ^{1}H NMR spectrum of **RA-NAP**.. 11

Figure S17. ^{13}C NMR spectrum of **RA-NAP** 12

Figure S18. HRESIMS spectrum of **RA-NAP** 12

N[5]*-((pyridin-2-ylamino)-L-glutamine* (**RA-PYD**)

Figure S19. ^{1}H NMR spectrum of **RA-PYD** 13

Figure S20. ^{13}C NMR spectrum of **RA-PYD** 13

Figure S21. HRESIMS spectrum of **RA-PYD**.. 14

N[5]*-((quinolin-2-ylamino)-L-glutamine* (**RA-2Q**)

Figure S22. ^{1}H NMR spectrum of **RA-2Q** 14

Figure S23. ^{13}C NMR spectrum of **RA-2Q**.. 15

Figure S24. HRESIMS spectrum of **RA-2Q** ... 15

N^5-((dimethylamino)-L-glutamine (**RA-DMe**)

Figure S25. ^{1}H NMR spectrum of **RA-DMe** ... 16

Figure S26. ^{13}C NMR spectrum of **RA-DMe** ... 16

Figure S27. HRESIMS spectrum of **RA-DMe** ... 17

N^5-(isopropylamino)-L-glutamine (**RA-IPr**)

Figure S28. ^{1}H NMR spectrum of **RA-IPr** ... 17

Figure S29. ^{13}C NMR spectrum of **RA-IPr** ... 18

Figure S30. HRESIMS spectrum of **RA-IPr** ... 18

N^5-morpholino-L-glutamine (**RA-Morp**)

Figure S31. ^{1}H NMR spectrum of **RA-Morp** ... 19

Figure S32. ^{13}C NMR spectrum of **RA-Morp** ... 19

Figure S33. HRESIMS spectrum of **RA-Morp** ... 20

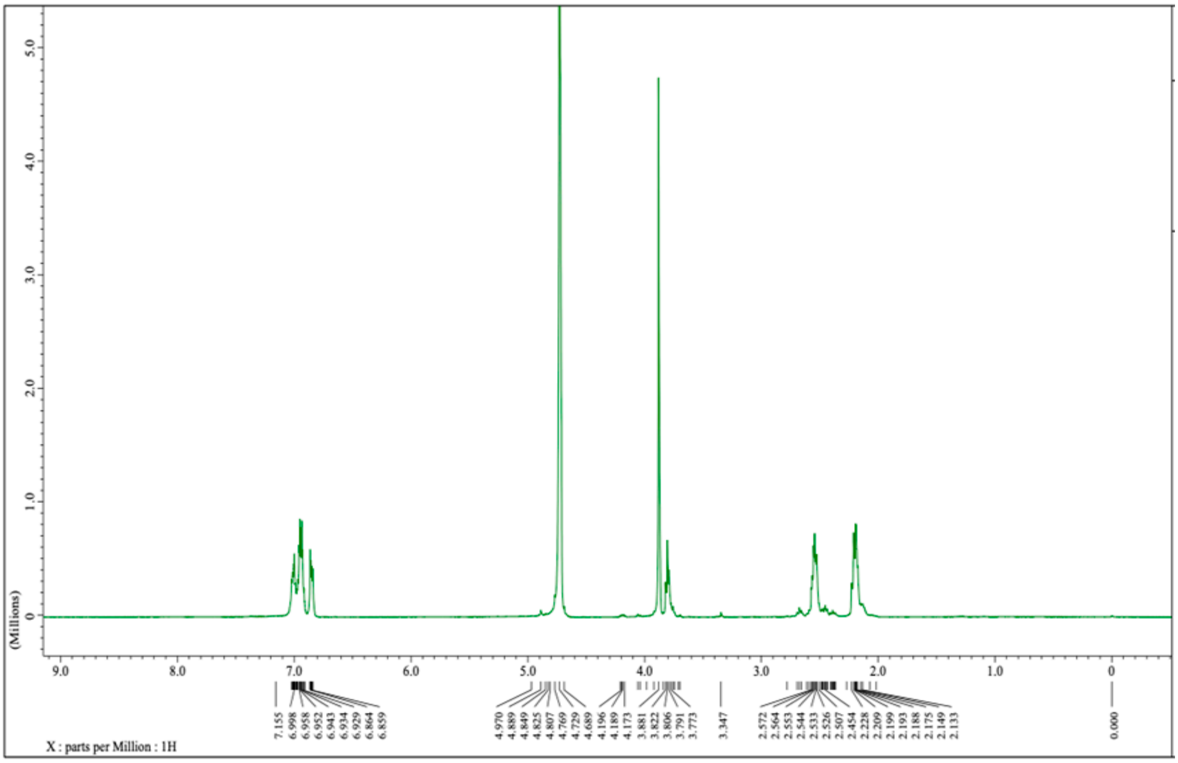

Figure S1: ^{1}H NMR (400 MHz) spectrum of **RA-2OMe**

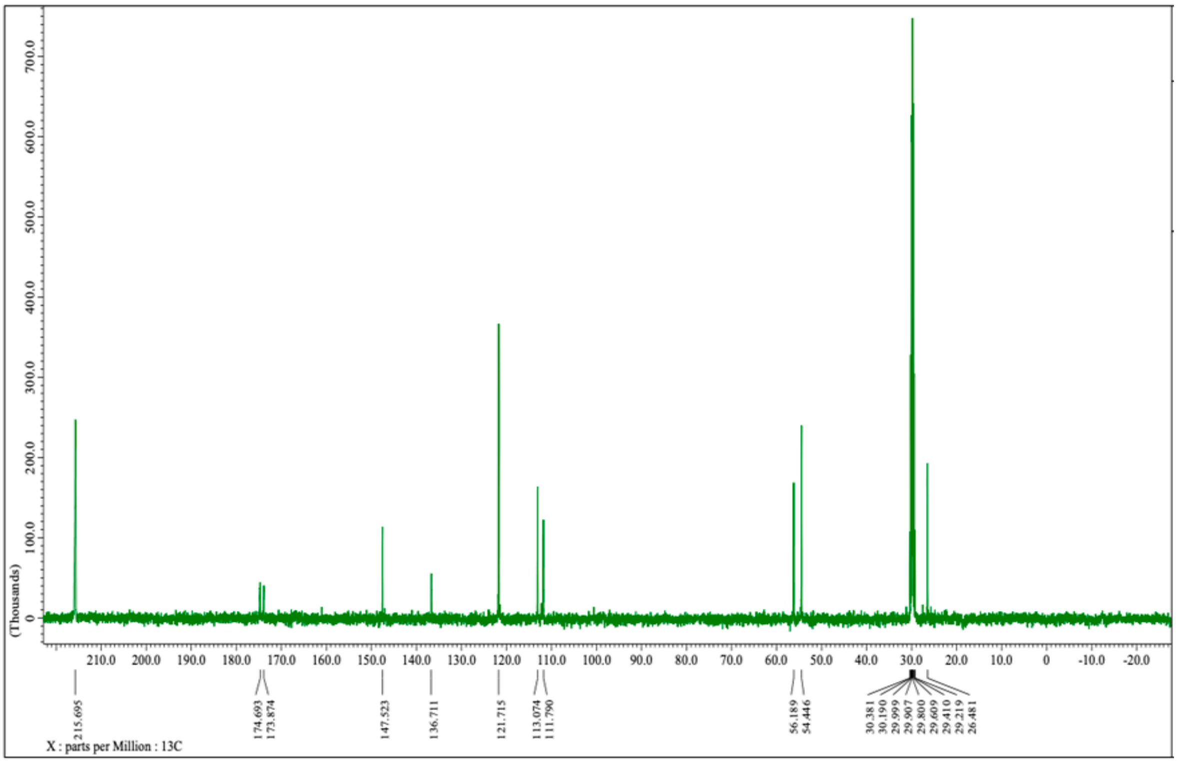

Figure S2: ^{13}C NMR (100 MHz) spectrum of **RA-2OMe**

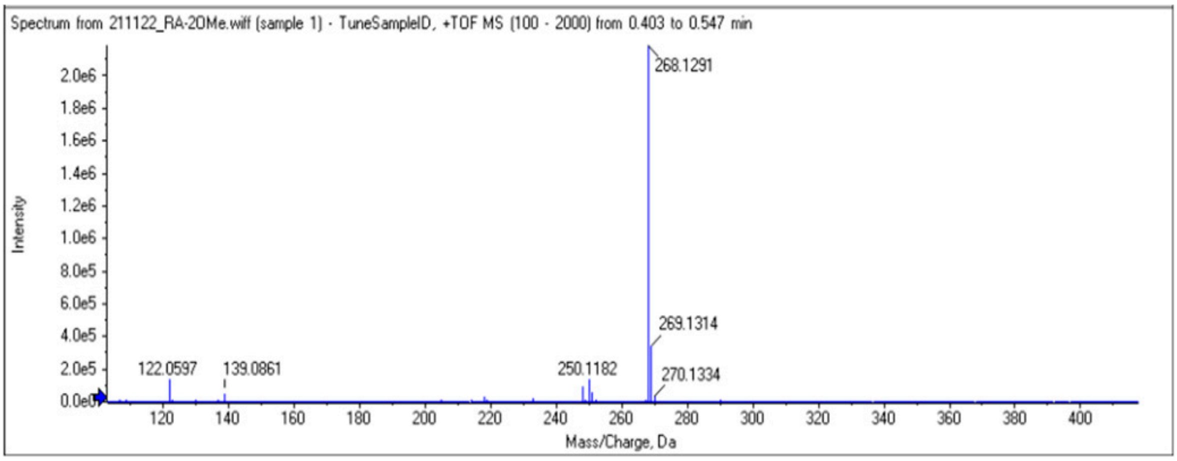

Figure S3: HRESIMS spectrum of **RA-2OMe**

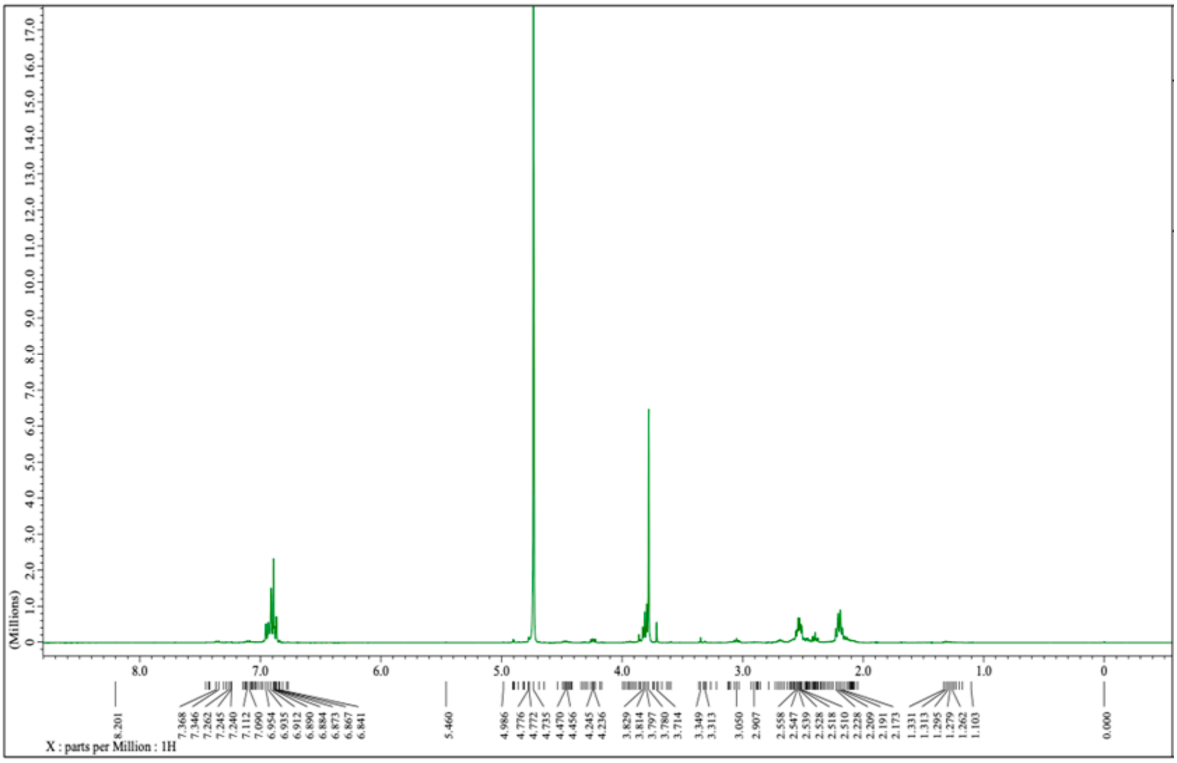

Figure S4: ^{1}H NMR (400 MHz) spectrum of **RA-4OMe**

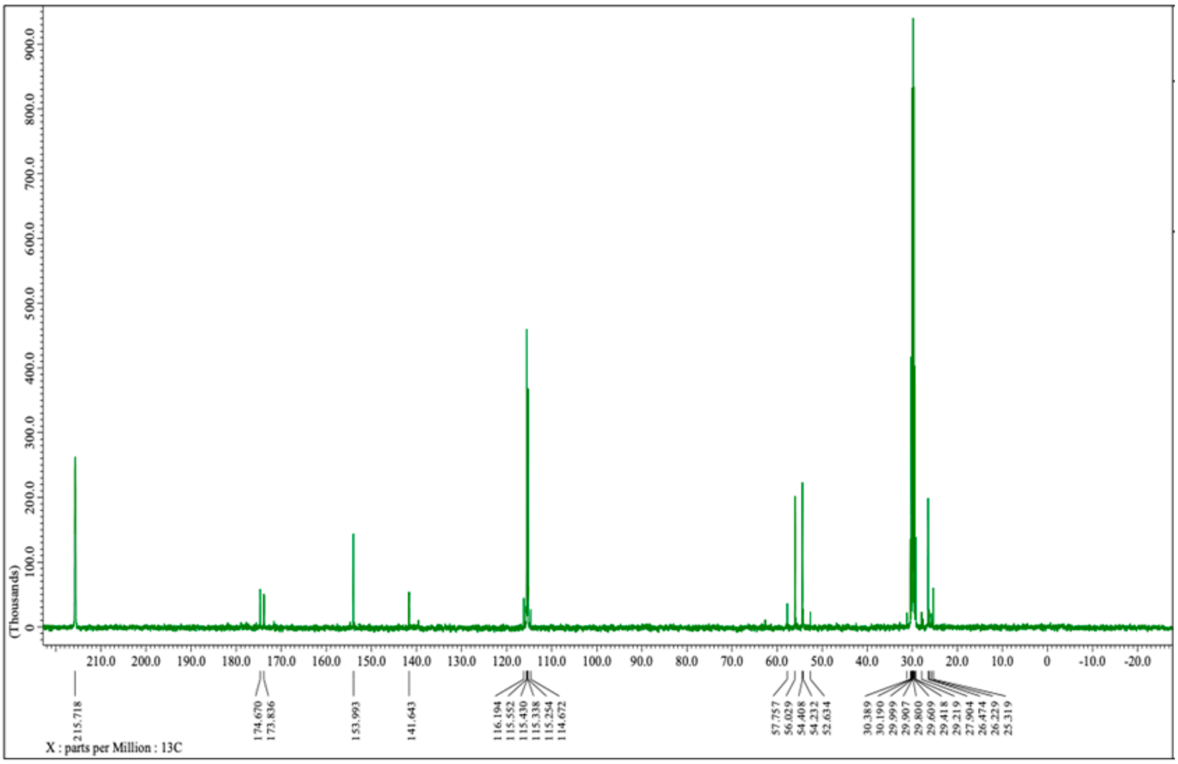

Figure S5: ^{13}C NMR (100 MHz) spectrum of **RA-4OMe**

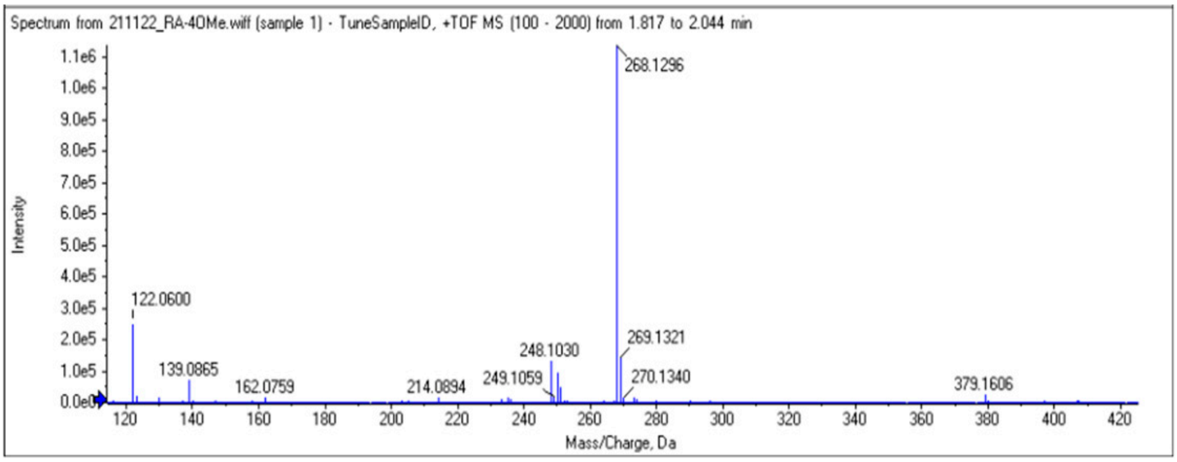

Figure S6: HRESIMS spectrum of **RA-4OMe**

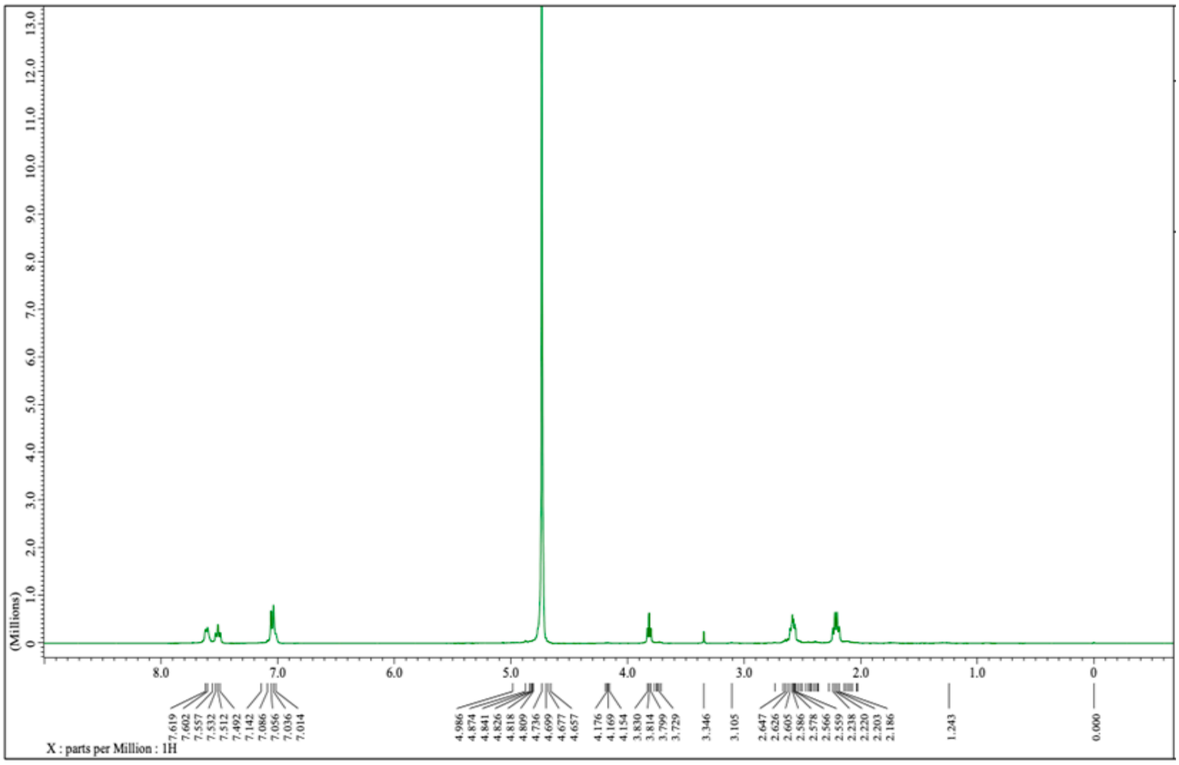

Figure S7: ^{1}H NMR (400 MHz) spectrum of **RA-2CF3**

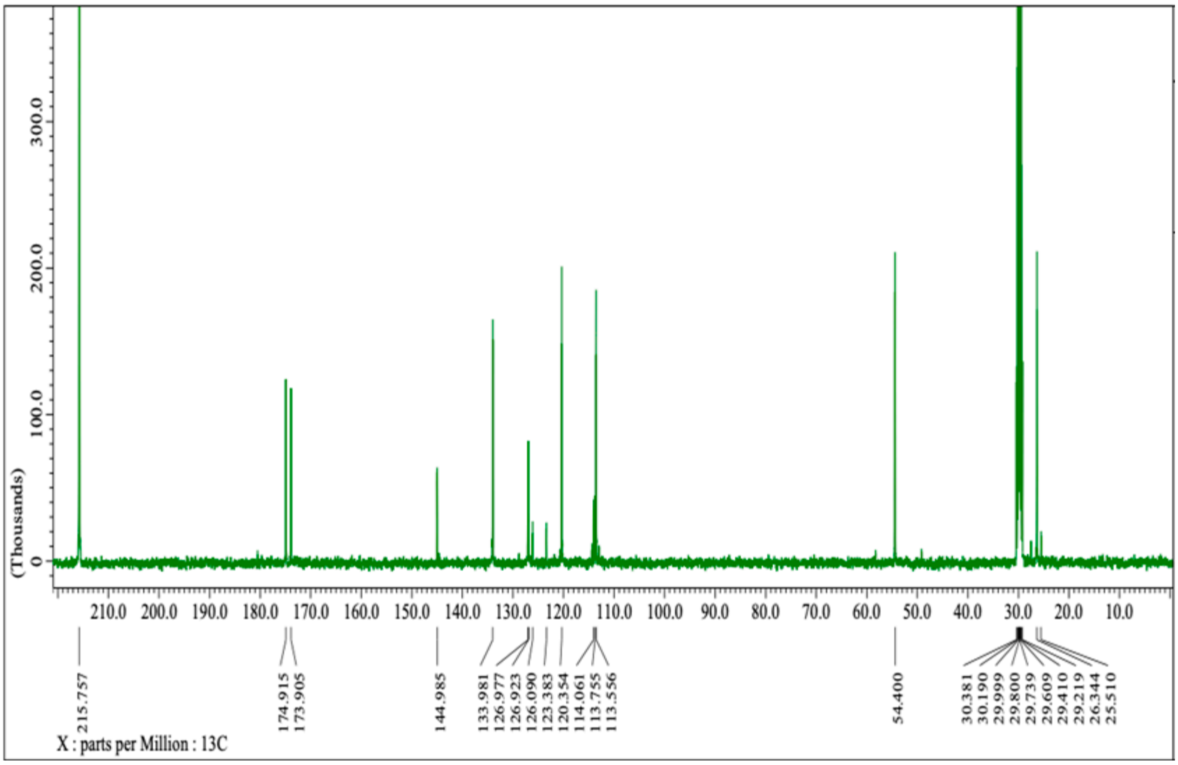

Figure S8: ^{13}C NMR (100 MHz) spectrum of **RA-2CF3**

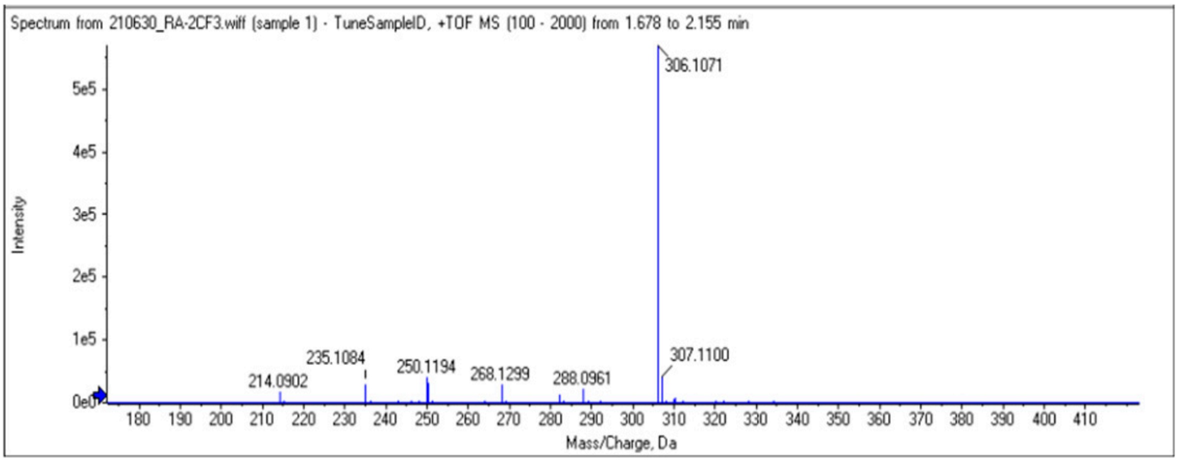

Figure S9: HRESIMS spectrum of **RA-2CF3**

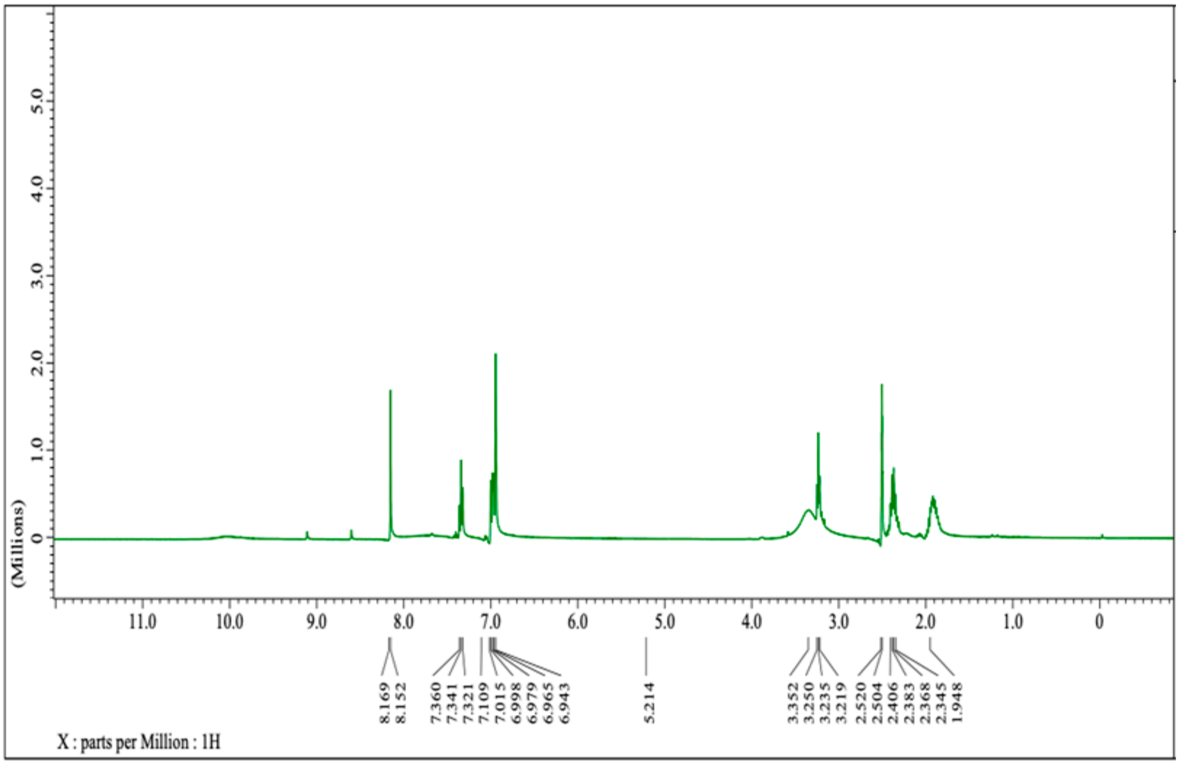

Figure S10: ^{1}H NMR (400 MHz) spectrum of **RA-3CF3**

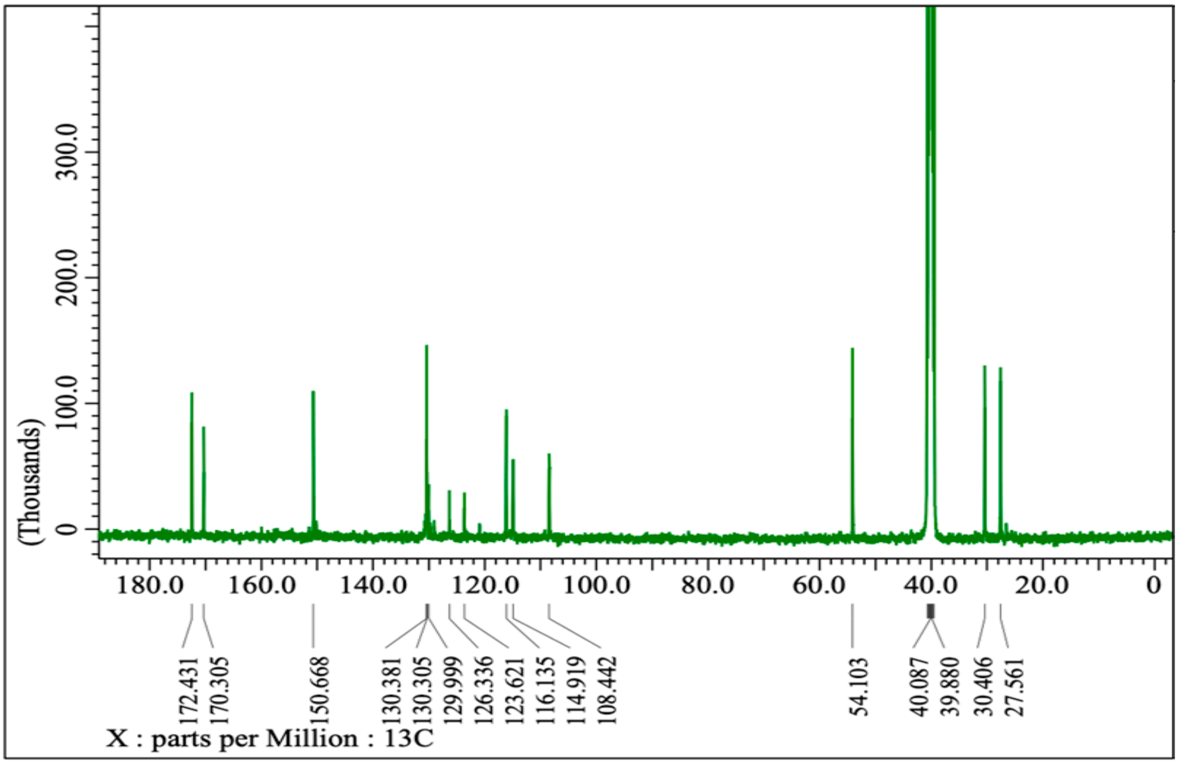

Figure S11: ^{13}C NMR (100 MHz) spectrum of **RA-3CF3**

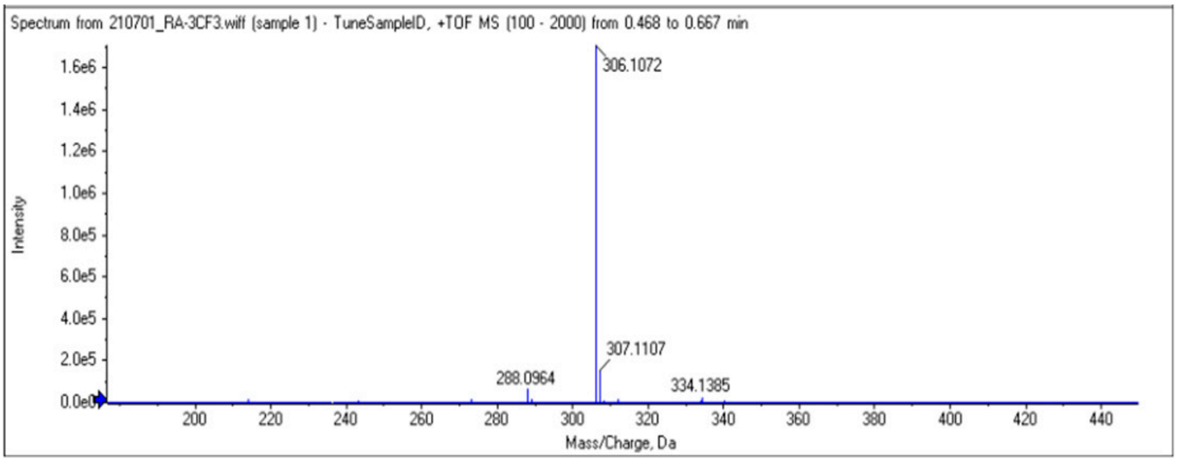

Figure S12: HRESIMS spectrum of **RA-3CF3**

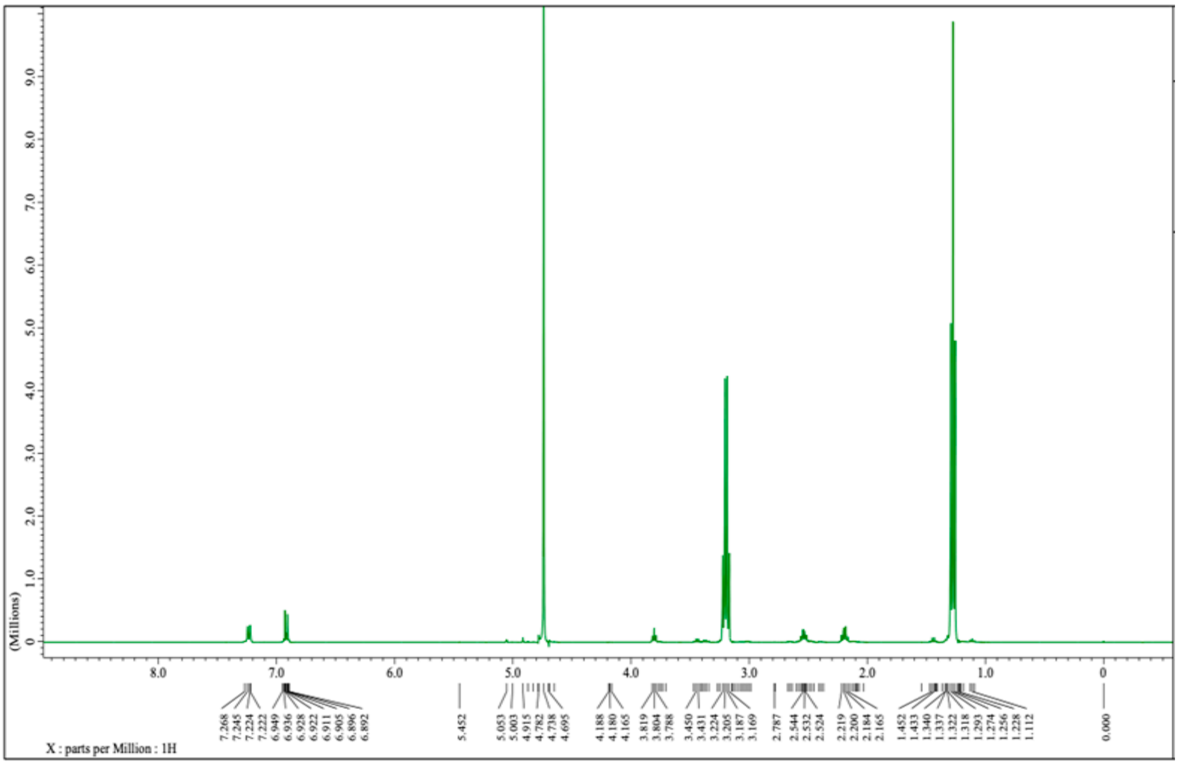

Figure S13: ^{1}H NMR (400 MHz) spectrum of **RA-4OCF3**

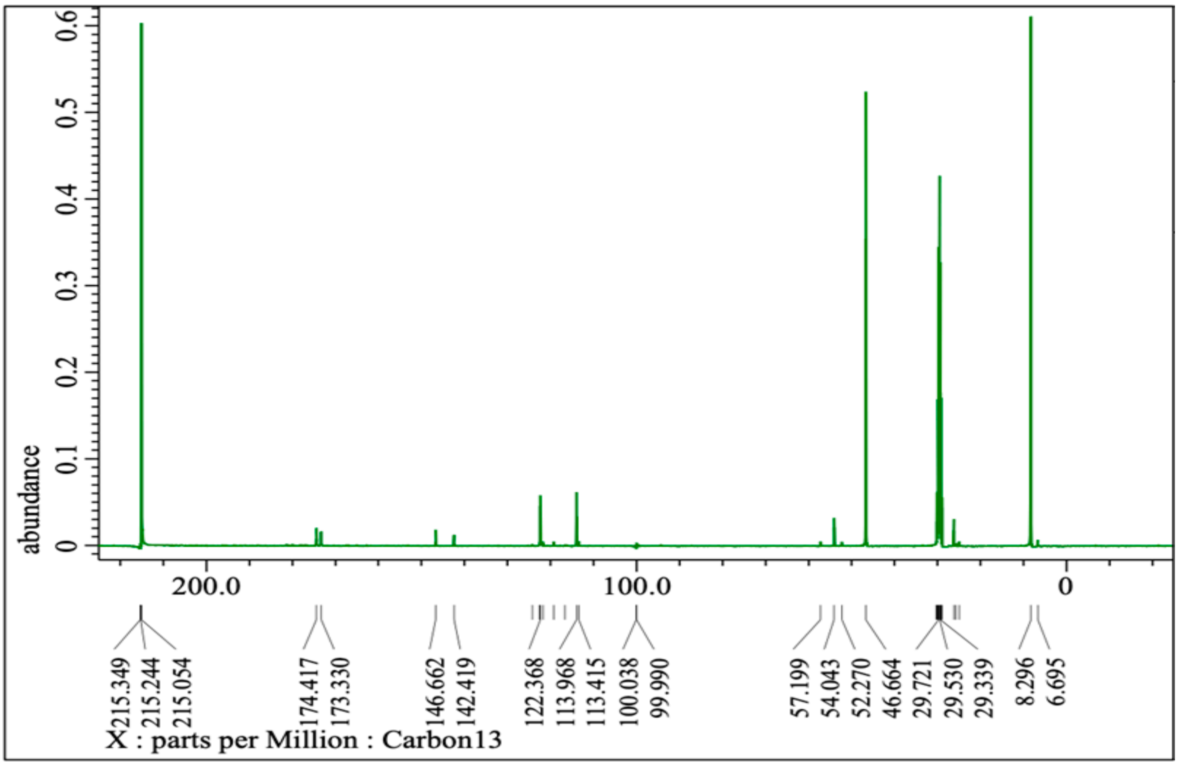

Figure S14: ^{13}C NMR (100 MHz) spectrum of **RA-4OCF3**

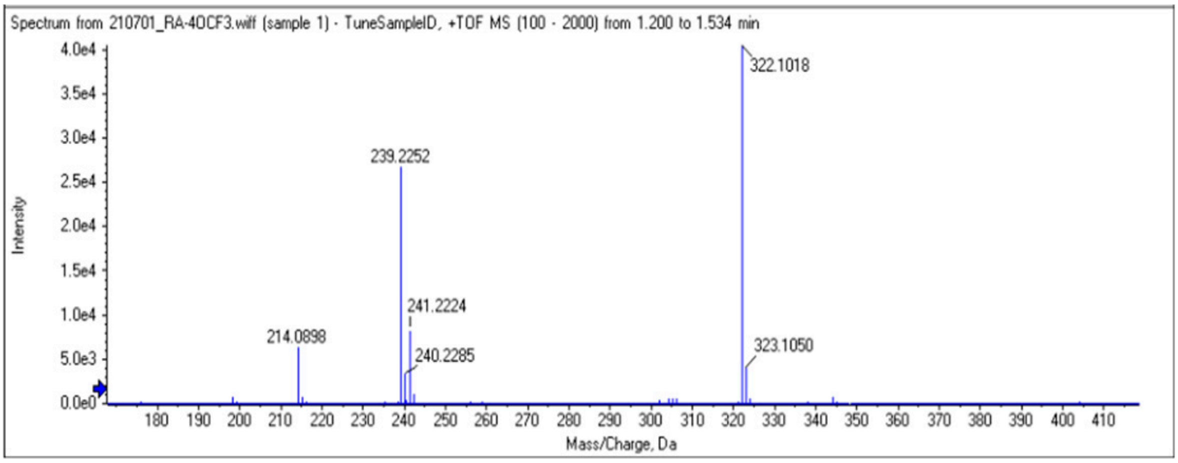

Figure S15: HRESIMS spectrum of **RA-4OCF3**

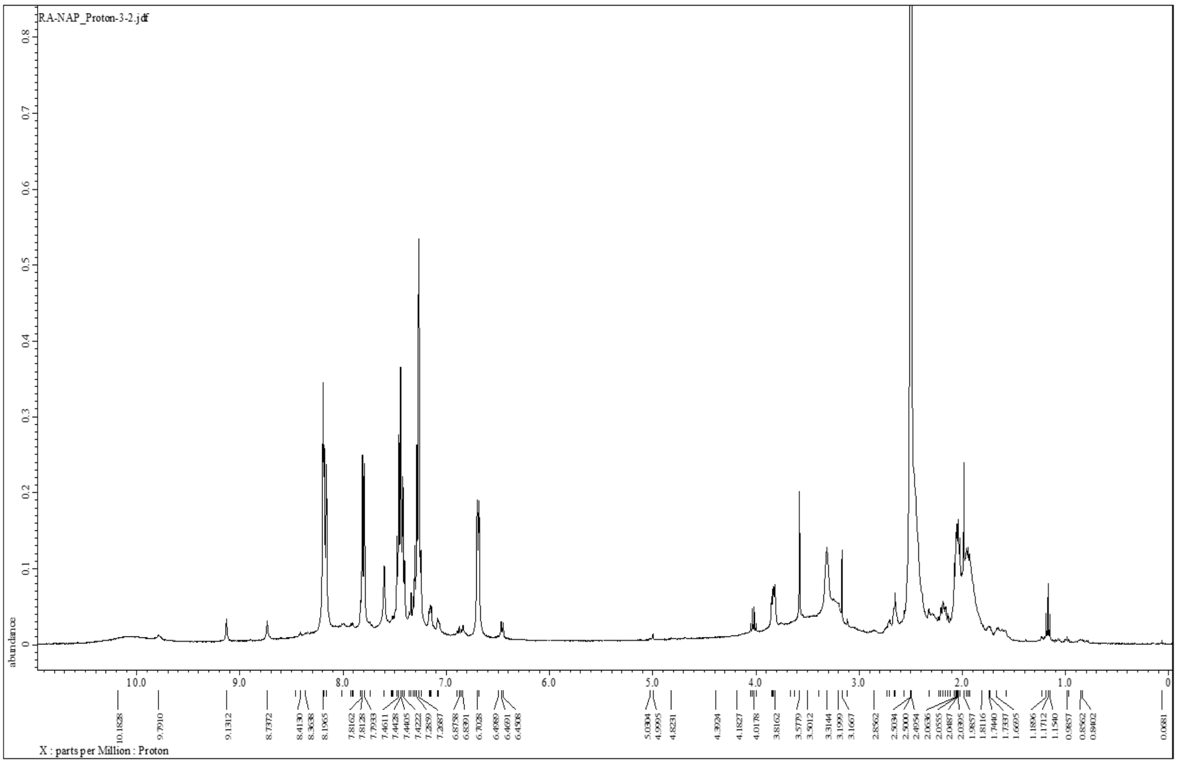

Figure S16: ^{1}H NMR (400 MHz) spectrum of **RA-NAP**

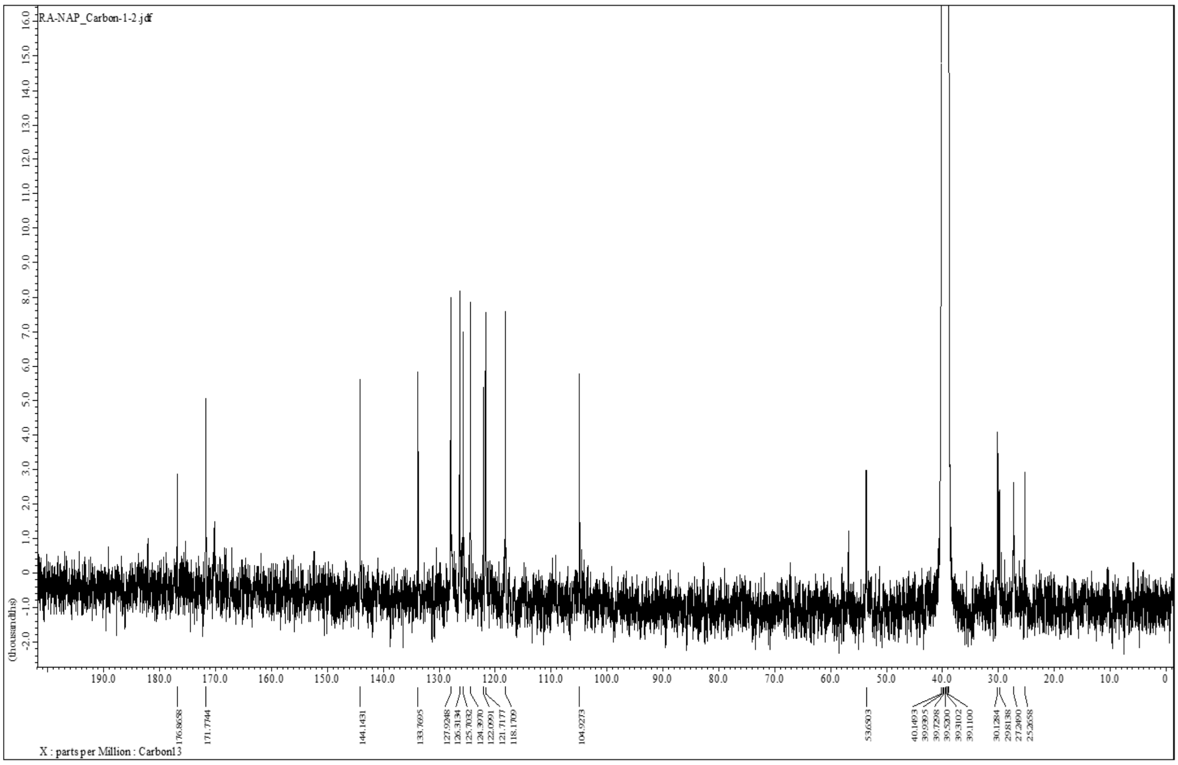

Figure S17: ^{13}C NMR (100 MHz) spectrum of **RA-NAP**

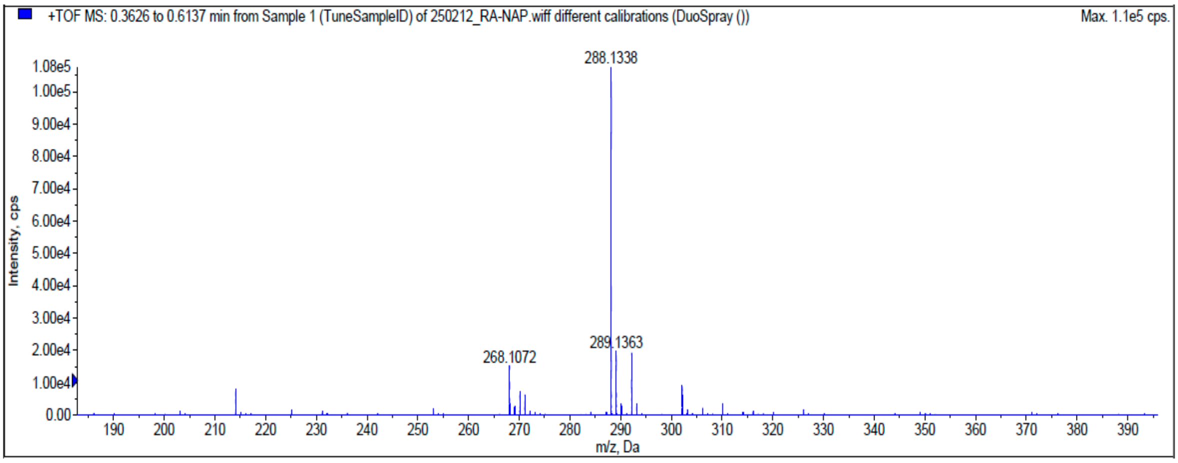

Figure S18: HRESIMS spectrum of **RA-NAP**

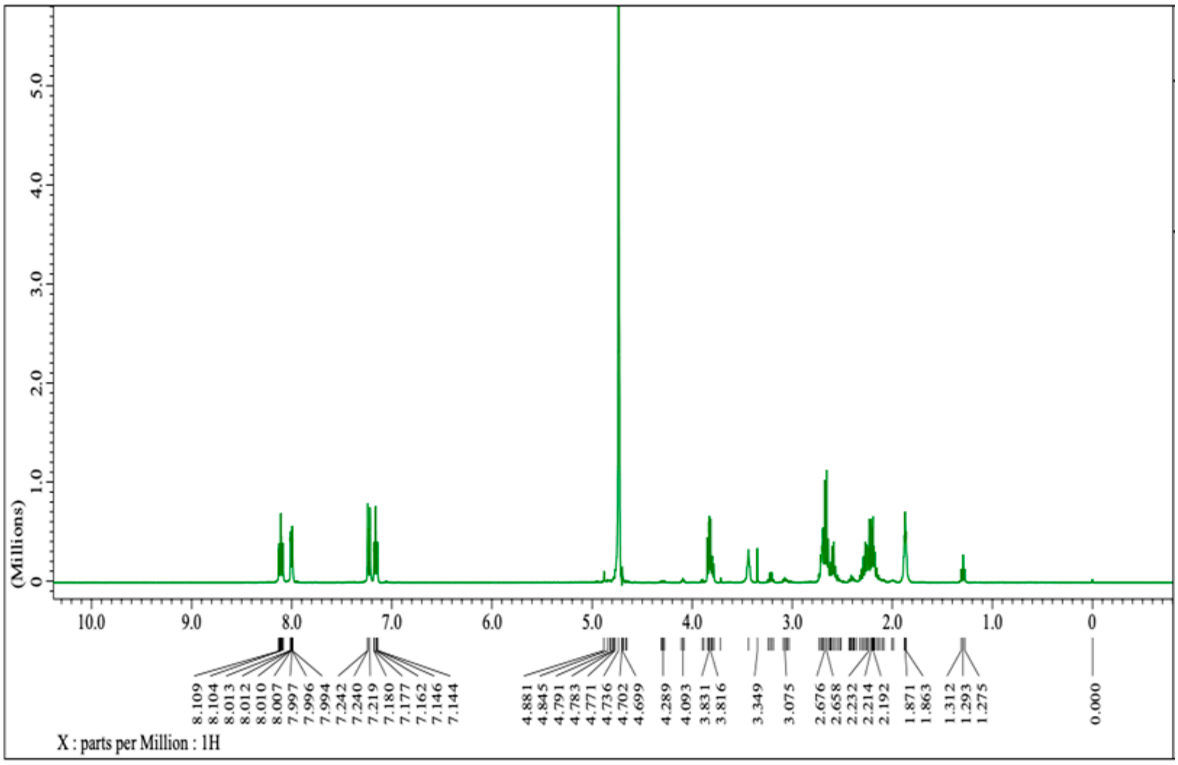

Figure S19: ^{1}H NMR (400 MHz) spectrum of **RA-PYD**

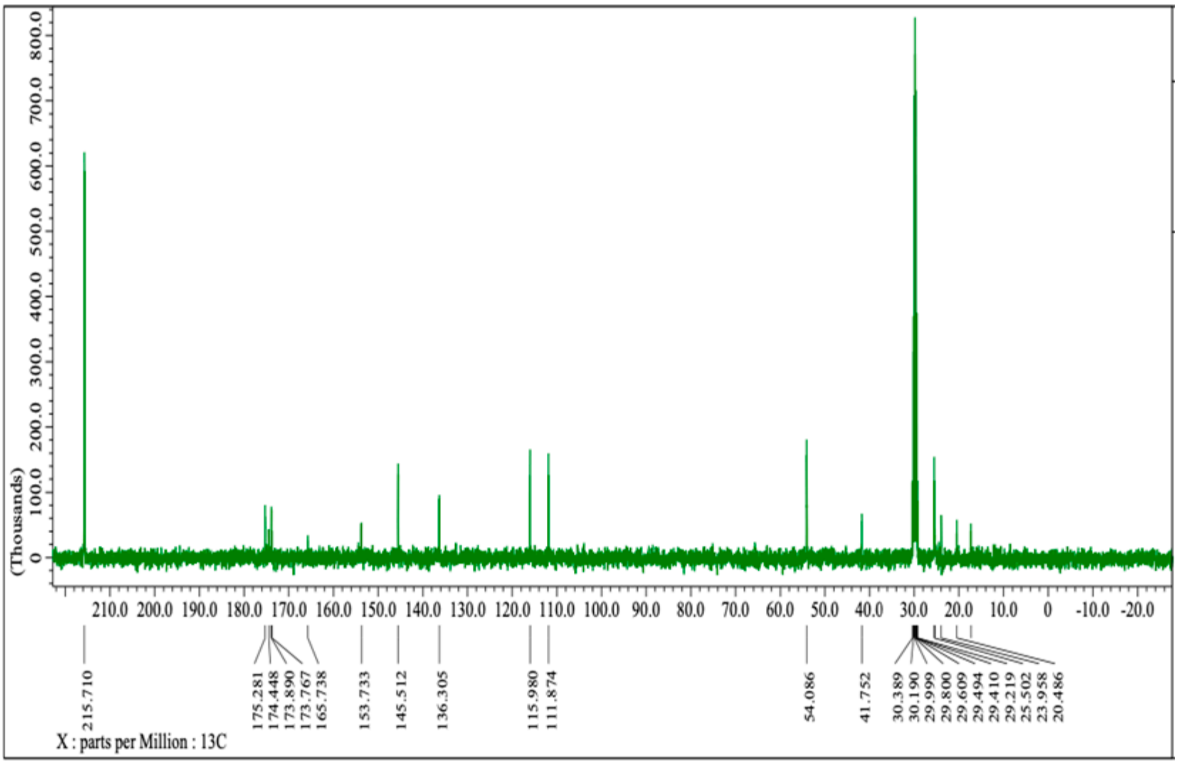

Figure S20: ^{13}C NMR (100 MHz) spectrum of **RA-PYD**

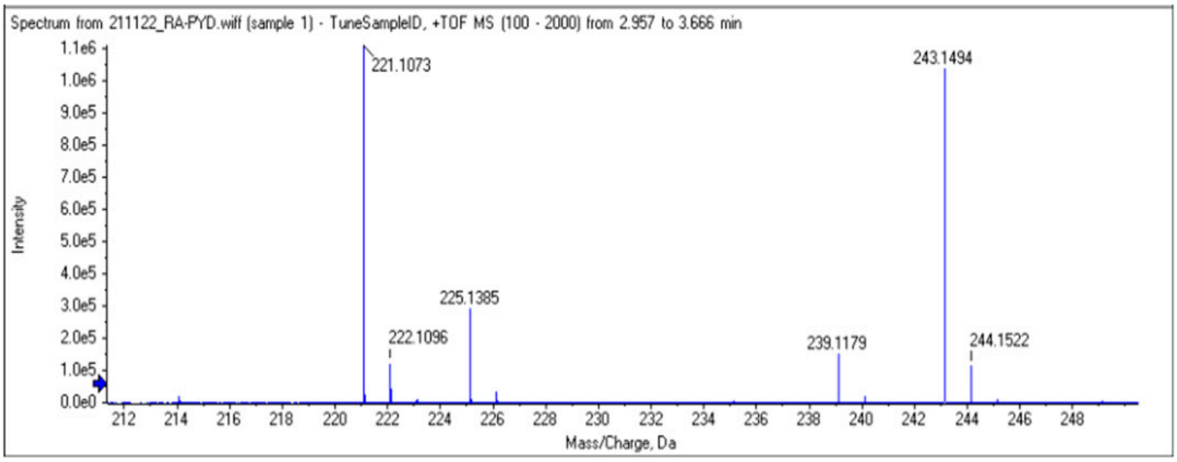

Figure S21: HRESIMS spectrum of **RA-PYD**

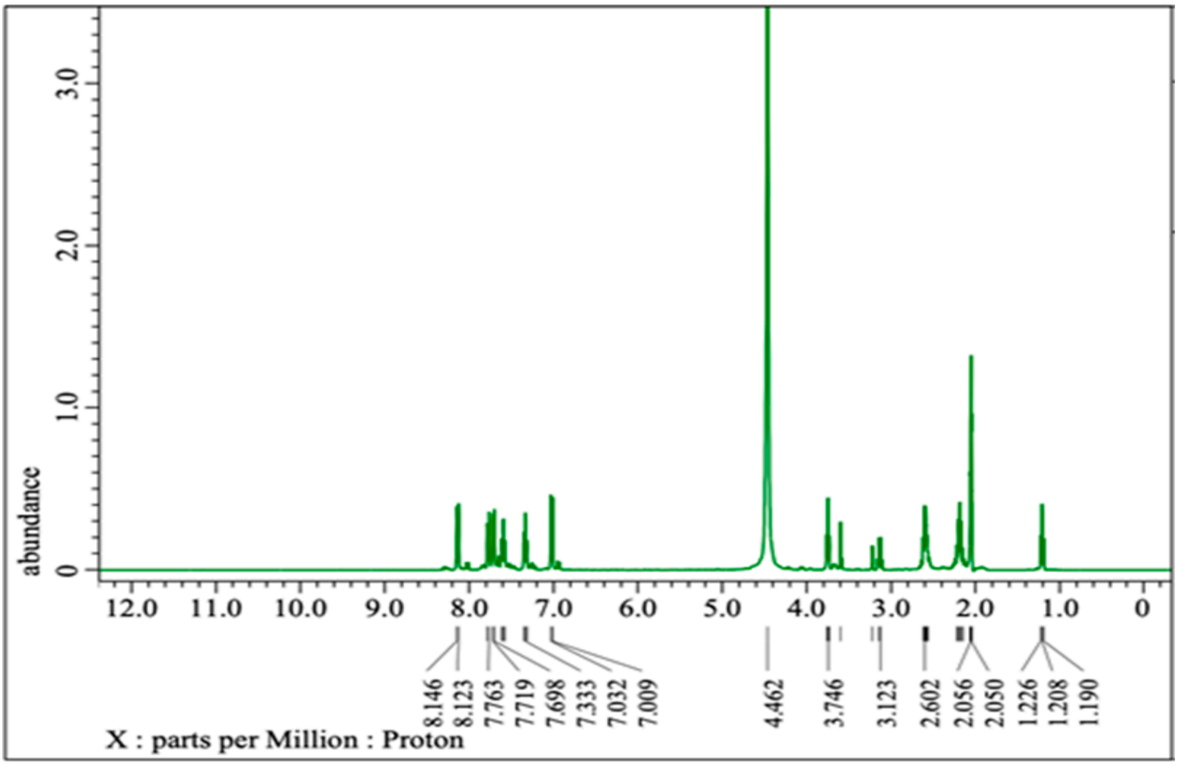

Figure S22: ^{1}H NMR (400 MHz) spectrum of **RA-2Q**

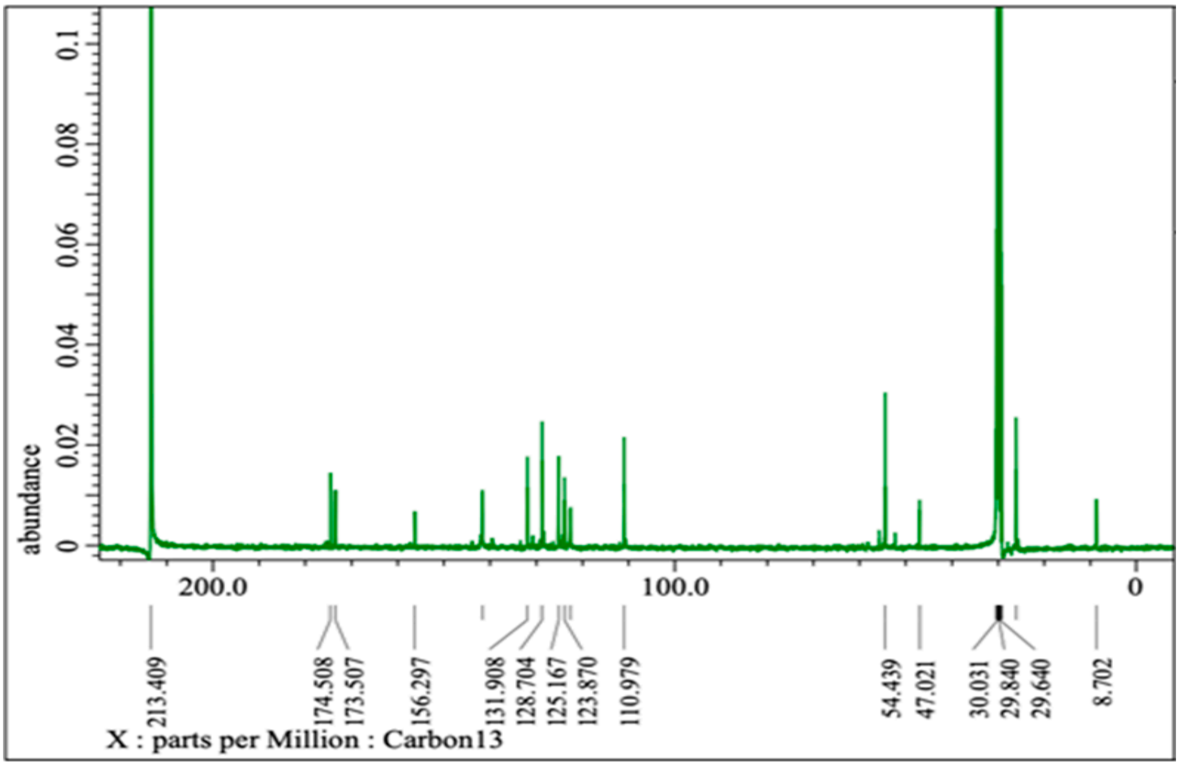

Figure S23: ^{13}C NMR (100 MHz) spectrum of **RA-2Q**

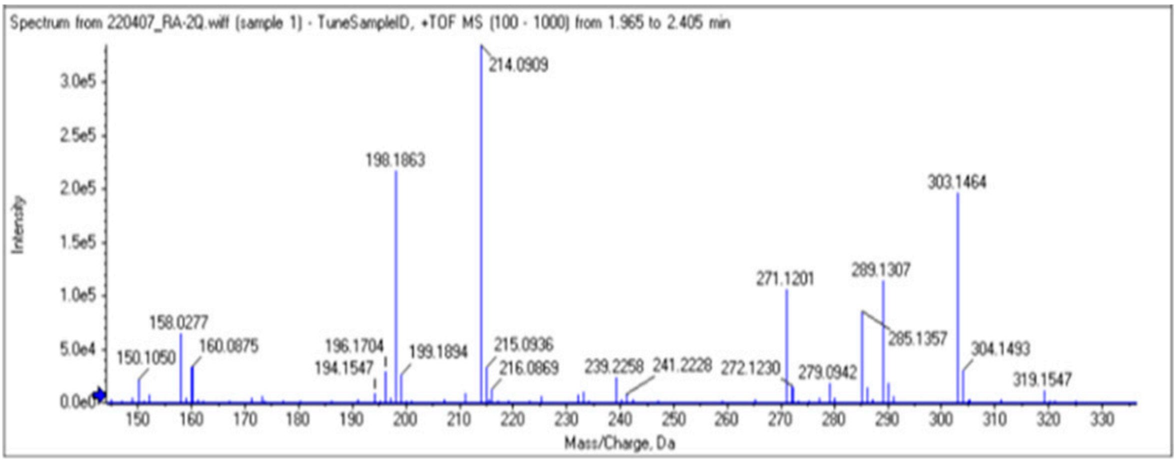

Figure S24: HRESIMS spectrum of **RA-2Q**

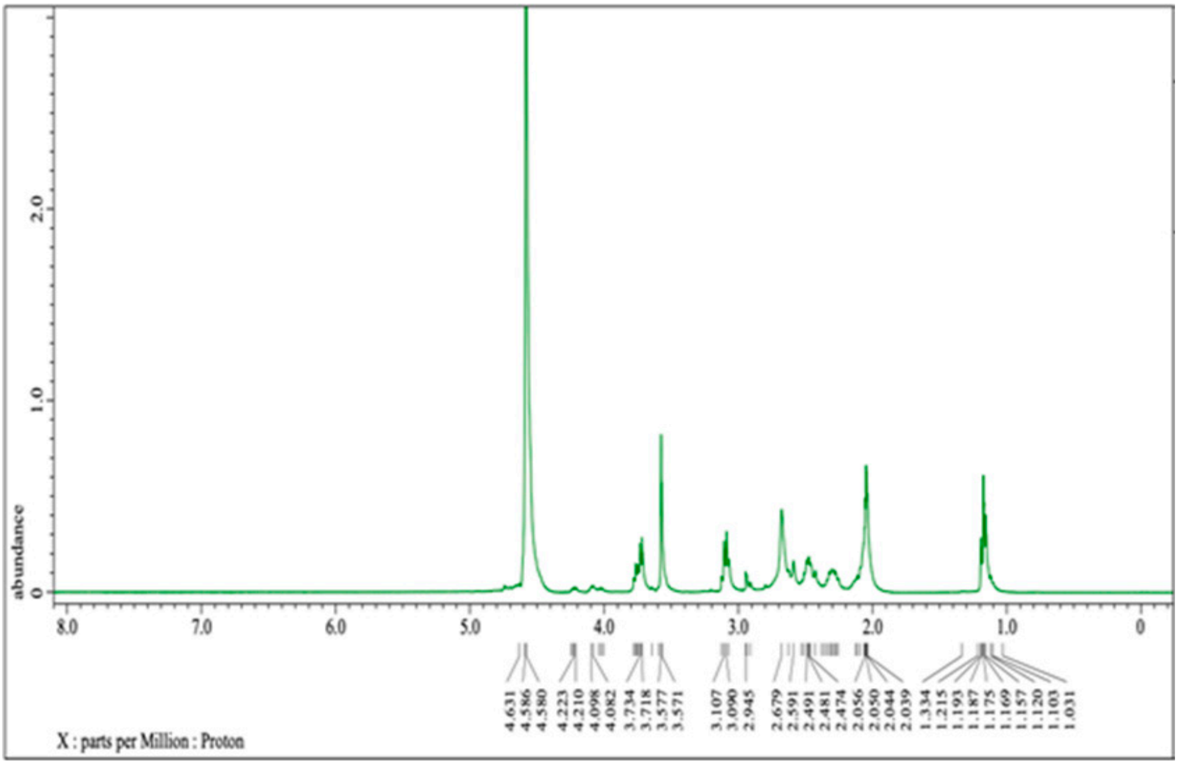

Figure S25: ^{1}H NMR (400 MHz) spectrum of **RA-DMe**

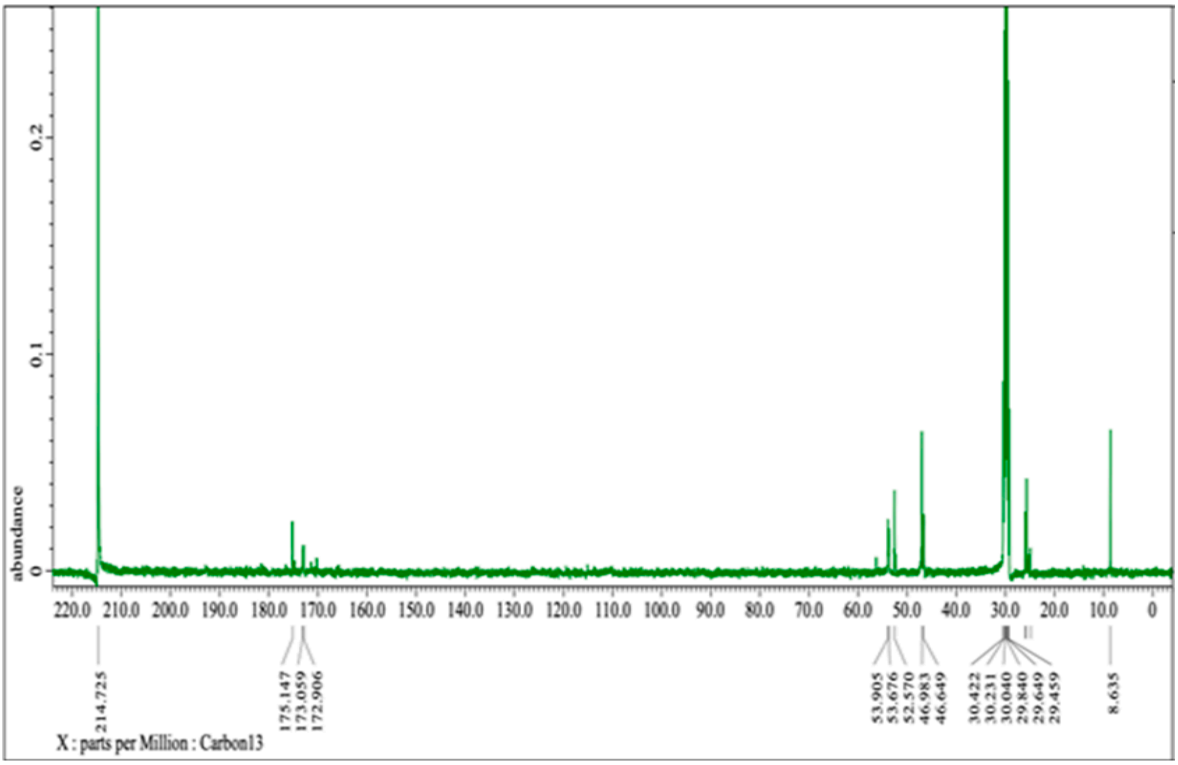

Figure S26: ^{13}C NMR (100 MHz) spectrum of **RA-DMe**

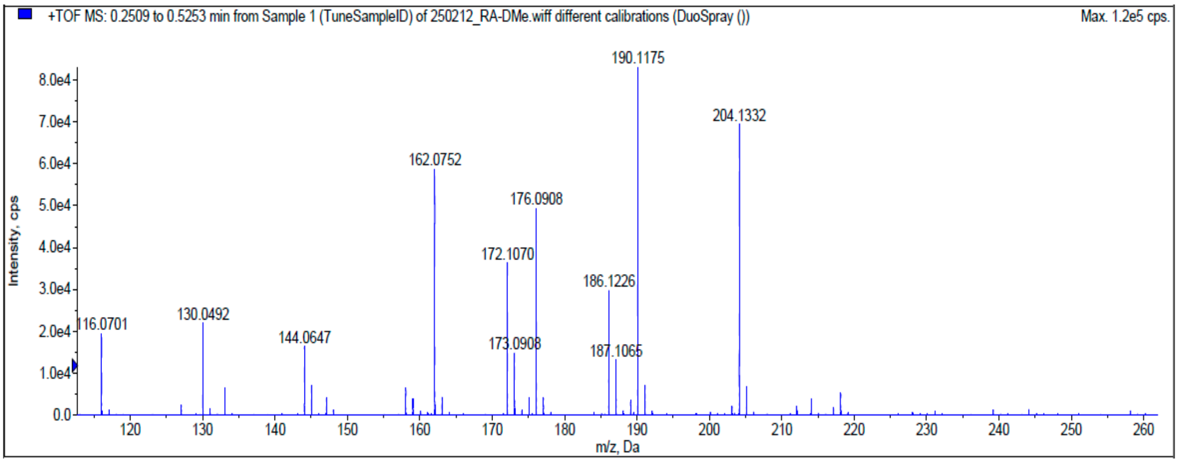

Figure S27: HRESIMS spectrum of **RA-DMe**

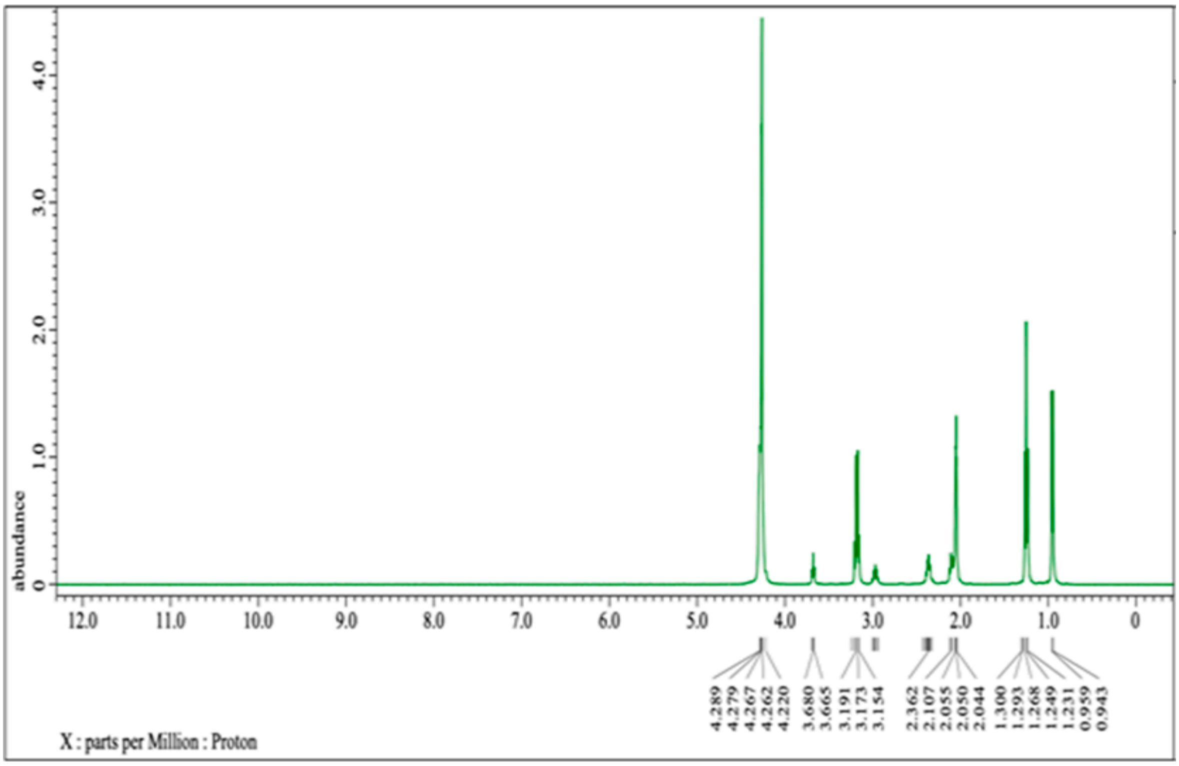

Figure S28: ^{1}H NMR (400 MHz) spectrum of **RA-IPr**

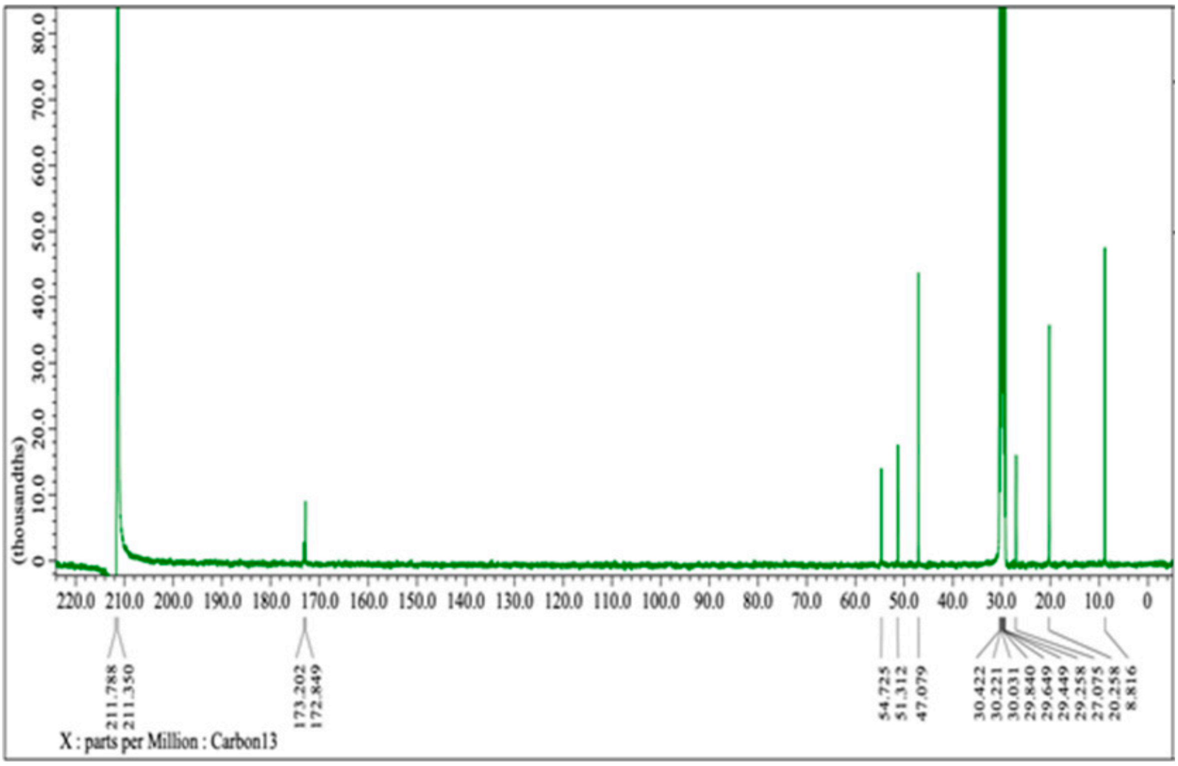

Figure S29: ^{13}C NMR (100 MHz) spectrum of **RA-IPr**

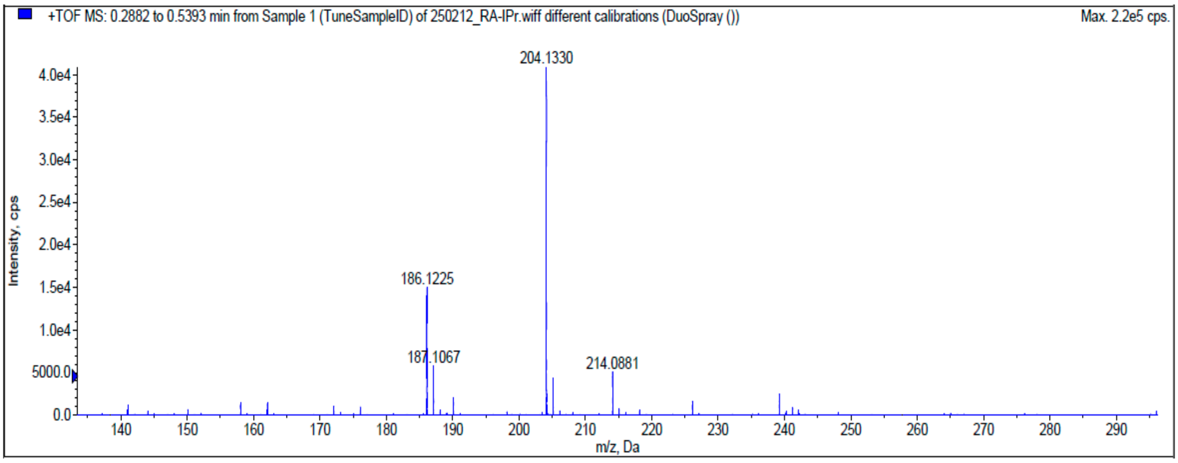

Figure S30: HRESIMS spectrum of **RA-IPr**

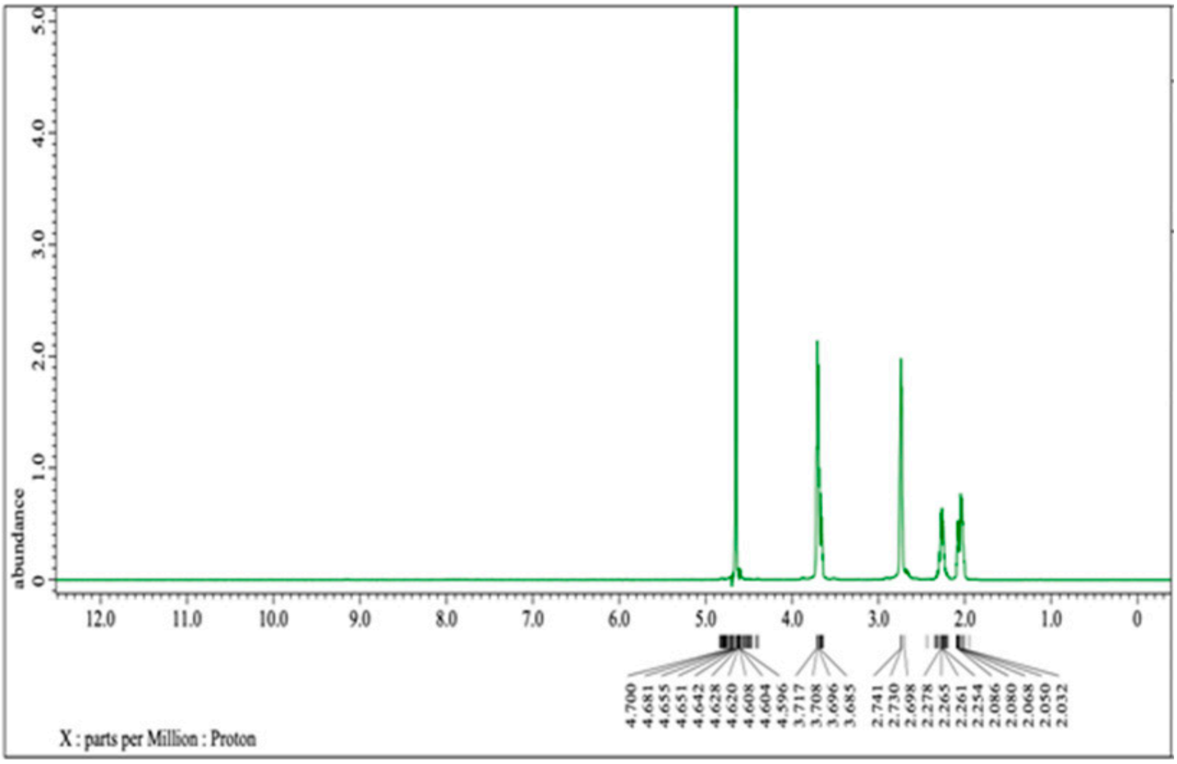

Figure S31: ^{1}H NMR (400 MHz) spectrum of **RA-Morp**

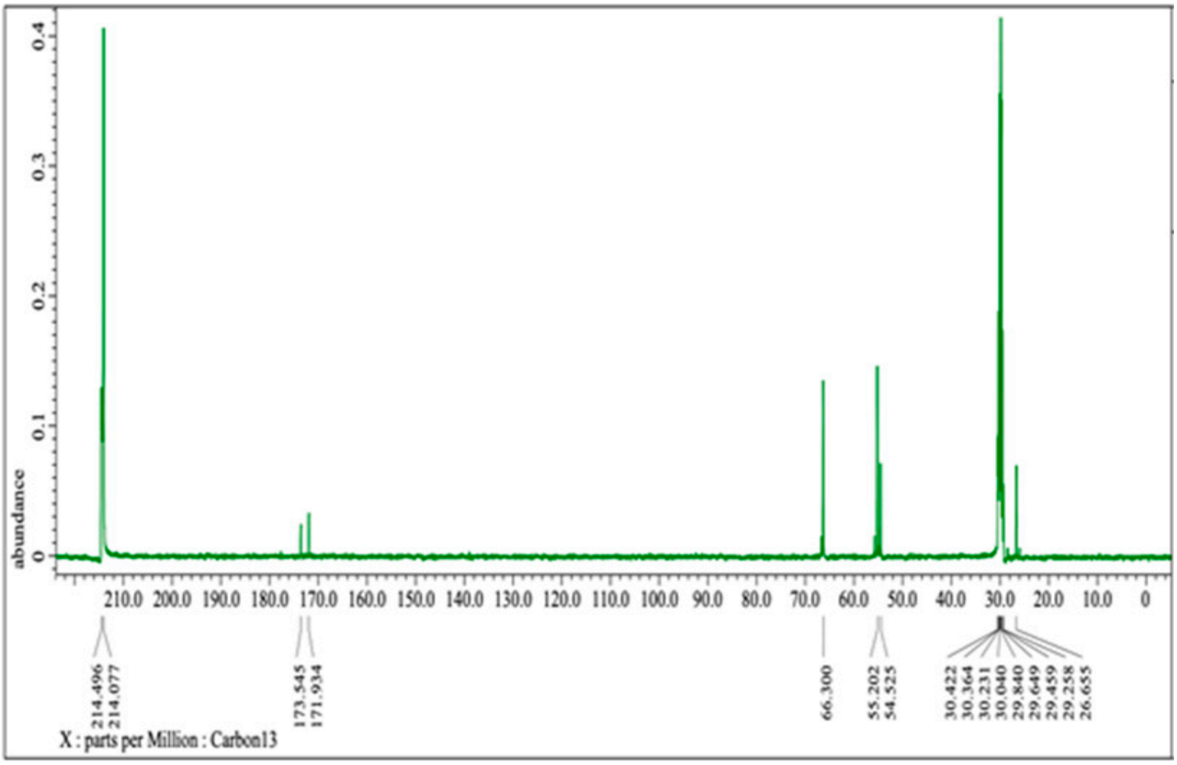

Figure S32: ^{13}C NMR (100 MHz) spectrum of **RA-Morp**

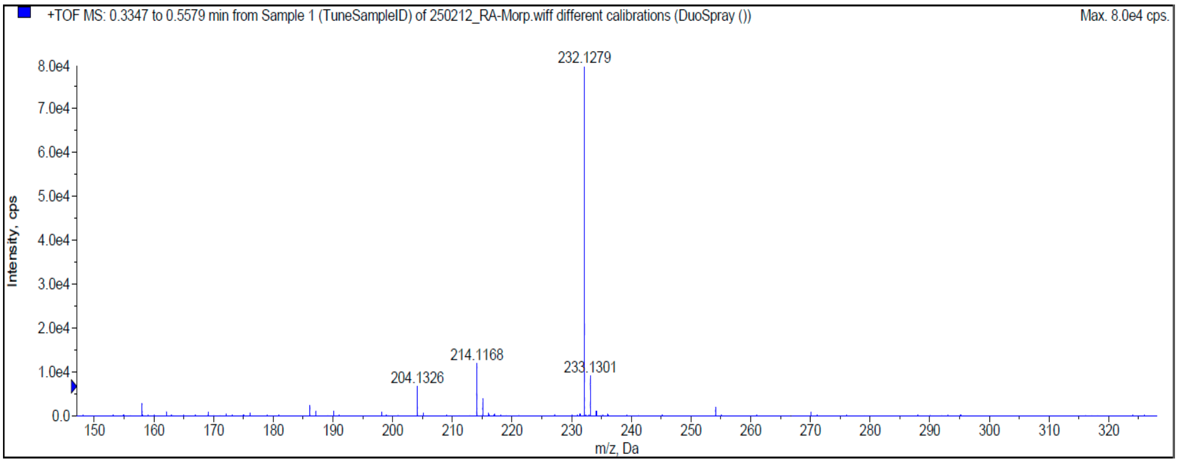

Figure S33: HRESIMS spectrum of **RA-Morp**
